# Pre-Administration of Berberine Exerts Chemopreventive Effects in AOM/DSS-Induced Colitis-Associated Carcinogenesis Mice via Modulating Inflammation and Intestinal Microbiota

**DOI:** 10.3390/nu14040726

**Published:** 2022-02-09

**Authors:** Jiaqiang Deng, Lili Zhao, Xieyong Yuan, Yan Li, Junyang Shi, Hua Zhang, Yuxuan Zhao, Liping Han, Huani Wang, Yan Yan, Hong Zhao, Haojie Wang, Fangdong Zou

**Affiliations:** Key Laboratory of Bio-Resources and Eco-Environment of Ministry of Education, College of Life Sciences, Sichuan University, Chengdu 610065, China; dengjiaqiang@stu.scu.edu.cn (J.D.); zllblue@126.com (L.Z.); 18375847648@163.com (X.Y.); 18054713505@163.com (Y.L.); sjy2016519954@163.com (J.S.); zhanghua8928@163.com (H.Z.); zyuxuan0424@163.com (Y.Z.); hanliping0204@163.com (L.H.); haohaowang7@163.com (H.W.); yanyancd40078@163.com (Y.Y.); zhh15528411935@163.com (H.Z.); cvn78f35c@gmail.com (H.W.)

**Keywords:** berberine, colitis-associated carcinogenesis, inflammation, intestinal microbiota, chemoprevention

## Abstract

Inflammatory activation and intestinal flora imbalance play an essential role in the development and progression of colorectal cancer (CRC). Berberine (BBR) has attracted great attention in recent years due to its heath-related benefits in inflammatory disorders and tumors, but the intricate mechanisms have not been fully elucidated. In this study, the effects and the mechanism of BBR on colon cancer were investigated in an azoxymethane (AOM)/dextran sodium sulfate (DSS)-induced colitis-associated carcinogenesis mice model. Our results showed that pre-administration of BBR showed a decrease in weight loss, disease activity index (DAI) score, and the number of colon tumors in mice, compared with the model group. The evidence from pathological examination indicated that the malignancy of intestinal tumors was ameliorated after pre-administration of BBR. Additionally, pre-administration with BBR suppressed the expression of pro-inflammatory factors (interleukin (IL)-6, IL-1β, cyclooxygenase (COX)-2 and tumor necrosis factor (TNF)-α) and the cell-proliferation marker Ki67, while expression of the tight junction proteins (ZO-1 and occludin) were increased in colon tissue. Moreover, the levels of critical pathway proteins involved in the inflammatory process (p-STAT3 and p-JNK) and cell cycle regulation molecules (β-catenin, c-Myc and CylinD1) exhibited lower expression levels. Besides, 16S rRNA sequence analysis indicated that pre-administration of BBR increased the ratio of Firmicutes/Bacteroidetes (F:M) and the relative abundance of potentially beneficial bacteria, while the abundance of cancer-related bacteria was decreased. Gavage with *Lactobacillus rhamnosus* can improve the anti-tumor effect of BBR. Overall, pre-administration of BBR exerts preventive effects in colon carcinogenesis, and the mechanisms underlying these effects are correlated with the inhibition of inflammation and tumor proliferation and the maintenance of intestinal homeostasis.

## 1. Introduction

Colorectal cancer (CRC) is one of the most frequent malignant tumors in the digestive tract with a complex etiology and high fatality rate, and it is correlated to lifestyle factors, including low physical activity, high-fat diets, alcohol consumption, smoking tobacco and sedentary behavior [1,2]. Several studies have demonstrated that inflammatory bowel disease (IBD) caused by chronic inflammatory conditions can increase the risk of CRC, such as ulcerative colitis (UC) and Crohn’s disease (CD) [3,4]. Multiple interrelated pathways were considered to be involved in the pathogenesis of IBD-associated CRC, including inflammatory response, oxidative stress, and intestinal microbiota [3,5]. Inflammatory response is driven by inflammatory cytokines, which are generated by tumor cells and the immune cells from tumor microenvironment. Among the secreted factors identified are prostaglandin (PG), nitric oxide (NO), cyclooxygenase (COX)-2, tumor necrosis factor-alpha (TNF-a), interleukin (IL)-1β and IL-6, which ultimately contribute to the development of tumors [6,7]. Accumulating evidence has demonstrated that inflammatory cytokines can enhance cancer cell growth rates and invasiveness through activating inflammatory signaling pathways, including NF-κB and STATs [8,9]. Additionally, Wnt/β-catenin signaling has been considered to be an indispensable player in tumorigenesis with its regulatory role on the inflammatory cascade [10].

Importantly, the interactions between resident micro-organisms and the intestinal tract are essential for maintaining gut homeostasis. Accordingly, alterations of the gut microbiota composition have been demonstrated to be involved in CRC progression [11]. Indeed, intestinal microbiota has been divided into three categories based on their effects in the intestinal tract, including physiologic bacteria, conditional pathogens, and pathogens [12]. Changes of the enteral and external environments can cause a diminish in the proportion of intestinal-dominant microbiota, while conferring a survival advantage upon pathogens or conditional pathogens [13,14]. Available evidence has demonstrated that the immune system acts as a crucial link in the interactions between gut microbiota and CRC. A dysbiotic microbial community with pro-carcinogenic features is considered to be a major contributor to colorectal carcinogenesis by influencing the inflammatory signals [15,16,17]. In this scenario, several potentially beneficial (*Lactobacillus*, *Bifidobacterium*, *Faecalibacterium prausnitzii*, *Roseburia*, and *Enterococcus*) and harmful (*Enterococcus*, Enterotoxigenic *bacteroides fragilis*, *Streptococcus*, and *Helicobacter*) bacterial species have been identified, and demonstrated to be important in CRC progression [11,12,13]. Therefore, the pathogenesis of CRC is closely correlated with the intestinal microbiota, and gut microbiota dysbiosis plays a significant role in the manifestation of CRC.

Continuing inflammatory conditions could account for the development of CRC, thus numerous anti-inflammatory agents have been regarded as potential chemopreventive agents, particularly food-derived and herb-derived multifunctional natural products [18,19,20,21]. Currently, numerous natural products have been extensively used in traditional and modern medicine due to the anti-inflammatory, anti-oxidative, anti-apoptotic, and anti-tumorigenic effects, which provide significant promise for cancer prevention and therapy, especially in inflammatory cancers [10]. However, the mechanisms underlying these effects exerted by natural products remain unclear. Therefore, much work should dedicated to exploring natural products, and simultaneously the precise mechanisms of therapeutic action need to be elucidated.

Berberine (BBR) is an isoquinoline alkaloid extracted from medicinal plants, such as Coptidis Rhizoma (*Huanglian*) and Cortex Phellodendri (*Huangbai*) [22]. In the last two decades, BBR has been investigated vigorously due to its manifold biological activities for anti-tumorigenic, anti-inflammatory, anti-oxidative, anti-microbial, anti-diabetic, and anti-hyperlipidemia properties [23,24,25,26]. Accordingly, the evidence from in vivo studies has confirmed that BBR exerts enormous therapeutic potential on various diseases, including cardiovascular diseases [27], metabolic diseases [28], inflammatory diseases [29] and cancers [30]. In terms of modern biomedical studies, the anti-cancer activities of BBR have been demonstrated, which were correlated to inhibit the proliferation, growth, and metastasis of tumors, including gastric cancer, pancreatic cancer, breast cancer, lung cancer, liver cancer and colorectal cancer [31]. Liu and colleagues found that BBR can inhibit the growth, migration/invasion of CRC cells via the COX-2/PGE2 mediated JAK2/STAT3 signaling pathway [32]. Other evidence indicated that BBR suppressed colon epithelial proliferation and tumorigenesis via AMPK dependent inhibition of mTOR and NF-κB signaling in mice [33]. Additionally, BBR has been demonstrated to modulate the tumor microenvironment by reinstating dysbiotic gut microbiota [34]. Thus, BBR exerts antineoplastic effects involved with multiple targets, and more investigations are required to reveal the complex mechanisms involved, especially in the anti-inflammatory effect and its mechanism in intestinal tract. Available data have suggested that the polypharmacology of BBR is in part explained by its role in modulation of the gut microbiota [35].

Although the effects of BBR on various cancers have been vigorous investigated, rare evidence has evaluated whether BBR pre-administration could exert the preventive effects in the development of cancers. In this study, BBR was administrated by the intragastric route prior to model induction, and we evaluated the preventive effects of BBR pre-administration on AOM/DSS-induced colitis and colorectal carcinogenesis based on body weight, disease activity index (DAI) score, and colon histology. Furthermore, the underlying mechanism was elucidated from the modulation of inflammation, cell proliferation, intestinal barrier function and microbiota.

## 2. Materials and Methods

### 2.1. Chemicals and Drugs

Berberine was obtained from Sangon Biotech Co., Ltd. (Shanghai, China). Azoxymethane (AOM) was purchased from Sigma Aldrich (St. Louis, MO, USA). Dextran sulfate sodium salt (DSS) was provided by Dalian Meilun Biotech Co., Ltd. (Dalian, China). The AIN-93M rodent diet was purchased from Trophic Animal Feed High-tech Co., Ltd. (Nantong, China). All antibodies used in this study, were obtained from Zhengneng Biotechnology Co., Ltd. (Chengdu, China).

### 2.2. Animals

C57BL/6 male mice (age: 7-weeks old, weight: 20 ± 2 g, provided by Chengdu Dashuo Biological Co., Ltd., Chengdu, China), were acclimated under standard temperature and humidity conditions with clean water and a standard laboratory rodent diet (AIN-93M rodent diet, Trophic Animal Feed High-tech Co., Ltd., Nantong, China), and kept in 12:12 light-dark conditions for 5 days. All animal experiments were approved by the Medical Ethics Committee of Medical College of Sichuan University (authorized facility No. 2022017001).

### 2.3. Berberine (BBR) Treatment and Experimental Design

**Figure 1:**
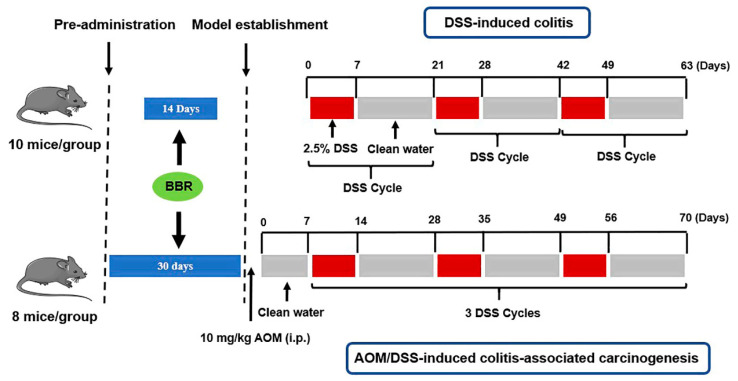
Animal experimental scheme.

To investigate the preventive effects of BBR on colorectal carcinoma, a DSS-induced colitis and AOM/DSS-induced colitis-associated carcinogenesis mice model was established [19,29,36]. Schematic representation of the animal experiment is shown in Figure 1.

DSS-induced colitis: Ulcerative colitis was induced chronically in mice by the administration of 2.5% DSS in the drinking for 1 week, and then normal drinking for 2 weeks (the 3 weeks period was defined as one DSS cycle, kept in 3 cycles). Animals were randomly divided into four groups (*n* = 10 mice/group), including the model group (DSS group), low-dose BBR group (7.5 mg/kg/day, DSS + BBR(L)), high-dose BBR group (15 mg/kg/day, DSS + BBR(H)) and control group. BBR was administrated by the intragastric route for 2 weeks prior to model induction in the experimental group.

AOM/DSS-induced colitis-associated carcinogenesis: Mice were injected intraperitoneally with AOM (10 mg/kg) at the initial stages of the experiment (day 0) and kept under normal drinking water for 1 week, and then three DSS cycles were implemented. The absolute control animals received sterile saline in both injections. Animals were randomly divided into four groups (*n* = 8 mice/group), as follows: the model group (AOM/DSS group), low-dose BBR group (7.5 mg/kg/day, AOM/DSS + BBR(L)), high-dose BBR group (15 mg/kg/day, AOM/DSS + BBR(H)) and control group. BBR was administrated by the intragastric route for 1 month prior to model induction in the experimental group.

Throughout the experimental period, the mice were weighed once a week. The disease activity index (DAI) score was used to monitor the severity of disease in the mice. Briefly, the changes in physiology (including spirit, activity, coat color, diet, and defecation) were observed and scored according to diagnostic criteria [36]. Then, 24 h after the final oral administration, mice were sacrificed by cervical dislocation after blood collection. The length of colorectal tissues and the number of tumors (diameter larger than or equal to 2 mm) in each group were recorded. In addition, fecal samples were collected in sterile conical tubes and stored at −80 °C.

### 2.4. Quantitative Real-Time Polymerase Chain Reaction (RT-qPCR)

The total RNA was extracted from colon tissue by using Trizol reagent (Invitrogen, USA), and cDNA was synthetized by using RNA reverse transcription kits (Takara, Japan). mRNA expression levels were examined on a CFX96 Touch Real-time PCR instrument (Bio-Rad, Richmond, CA) using a SYBR Green PCR Master Mix (Takara, Japan). β-actin was used as an internal control to normalize target genes transcription, and their mRNA relative expression was calculated by the 2^−ΔΔCt^ method. The primer sequences used were as follows: IL-1β (Forward: 5′-TGG ACC TTC CAG GAT GAG GAC A-3′; Reverse: 5′-GTT CAT CTC GGA GCC TGT AGT G-3′), IL-6 (Forward: 5′-TAC CAC TTC ACA AGT CGG AGG C-3′; Reverse: 5′-CTG CAA GTG CAT CAT CGT TGT TC-3′), TNF-α (Forward: 5′-GGT GCC TAT GTC TCA GCC TCT T-3′; Reverse: 5′-GCC ATA GAA CTG ATG AGA GGG AG-3′), COX-2 (Forward: 5′-GCG ACA TAC TCA AGC AGG AGC A-3′; Reverse: 5′-AGT GGT AAC CGC TCA GGT GTT G-3′). β-actin (Forward: 5′-GTC GTA CCA CAG GCA TTG TGA TGG-3′; Reverse: 5′-GCA ATG CCT GGG TAC ATG GTG G-3′).

### 2.5. Histopathological Analysis

Colon tissues in each group were fixed with 10% neutral formalin, subsequently embedded into paraffin, and cut into 5 μm-thick sections for staining with hematoxylin-eosin (H&E). Finally, the slides were examined under a light microscope.

### 2.6. Immunohistochemistry

After deparaffinization and rehydration, heat-induced antigen retrieval was implemented on the colon tissue sections under microwave irradiation. To block endogenous peroxidase activity, the slides were exposed to 3% H_2_O_2_ for 10 min at room temperature. Then, the sections were incubated overnight with primary antibodies at 4 °C prior to incubation with secondary antibodies for 1 h at room temperature. After that, the chromogenic reaction was carried out by using a DAB reagents (Sangon Biotech Co., Ltd., Shanghai, China), and the slides were counterstained with hematoxylin.

### 2.7. Western Blotting Assay

Frozen colon tissues were ground up in liquid nitrogen before harvesting proteins by using RIPA lysis buffer. Samples were centrifuged at 15,000 r/min for 10 min at 4 °C and stored at −80 ℃ after the determination of protein concentrations. For Western blotting, protein samples were separated by 10% sodium dodecyl sulfate (SDS) polyacrylamide gel electrophoresis and transferred onto a polyvinylidene fluoride (PVDF) membrane, which was then blocked with 5% skimmed milk for 1 h at room temperature. Afterward, the blots were incubated with primary antibodies at 4 °C overnight. The specific primary antibodies used were as follows: p-JNK (381100; dilution, 1:1000), p-STAT3 (381552; dilution, 1:5000), β-catenin (383616; dilution, 1:1000), CyclinD1 (382442; dilution, 1:2000), and C-Myc (380784; dilution, 1:1000). After washing with TBST, the membranes were incubated with horseradish peroxidase (HRP)-conjugated secondary antibody for 1 h at room temperature, and were then visualized by using the ECL detection system (Millipore, MA, USA). The band density was quantified using ImageJ software for each group and normalized with β-actin.

### 2.8. The 16S rRNA Microbial Community Analysis

Fecal samples were collected from five randomly selected mice in each group, the effects of BBR on the gut microbial communities were determined by 16S rRNA gene analysis. The sequencing work was conducted in Sinotech Genomics Co., Ltd. (Shanghai, China). Specifically, total bacterial DNA was extracted by using the PowerSoil DNA Isolation Kit (Mobio Labs, Solana Beach, USA). The V3–V4 region of the bacterial 16S rRNA gene was amplified with the primers (338F: 5′-ACT CCT ACG GGA GGC AGC AG -3′; 806R: 5′-GGA CTA CHV GGG TWT CTA AT-3′). The PCR was performed in a total volume of 20 µL, consisting of 15 μL of TransStart^®^ FastPfu PCR Master Mix (TransGen Biotech Co., Ltd., Beijing, China), 5 μM of both forward and reverse primers, 0.2 µL of bovine serum albumin (BSA), and 10 ng of template DNA. PCR was performed under the following conditions: an initial denaturation at 95 °C for 3 min, then 27 cycles at 95 °C for 30 s, 55 °C for 30 s, and 72 °C for 45 s, with a final extension step of 72 °C for 10 min. PCR products were purified using the AxyPrep DNA Gel Extraction Kit (Axygen Biosciences, Union City, CA, USA) prior to quantification by QuantiFluor^TM^-ST (Promega Corporation, Madison, WI, USA).

The purified amplicons were pooled in equimolar concentrations and sequenced using the Illumina MiSeq PE300 platform (Illumina, San Diego, CA, USA). The raw data (fastq format) were quality-filtered by Trimmomatic and merged by FLASH [37]. High-quality reads were selected and all of the effective reads were clustered into operational taxonomic units (OTUs) using the Usearch pipeline (http://www.drive5.com/usearch/ (accessed on 31 August 2020)) with 97% similarity cutoff. The representative sequences of OTUs were compared with the Silva 16S rRNA Database (release 123) using the RDP Classifier algorithm (v 2.11. https://sourceforge.net/projects/rdp-classifier/ (accessed on 31 August 2020)). In order to analyse the alpha diversity, the α-diversity values of the samples were calculated using Mothur (v1.33.3) software, and corresponding rarefaction curves were generated using R (v4.1.1) software to judge whether the data generated were sufficient to cover all species in the community. The ACE index and Shannon index were performed by R software (v4.1.1) to reflect the species richness and diversity of the community, respectively. Additionally, the beta diversity analysis was performed by QIIME software (v1.80), and the weighted UniFrac principal component analysis (PCoA) was employed to evaluate the similarity and otherness of sample community composition.

### 2.9. Identification of Potential Probiotics Isolated from Fecal Samples

Fecal samples were inoculated into a de Man Rogosa Sharpe (MRS) broth. After 28 h incubation, suspensions were also distributed on MRS agar plates with a spreader and cultured in a incubator (culture conditions: 37 °C, 5% CO_2_, 48 h). After that, colonies with different morphology were randomly selected and preliminarily screened by morphological and phenotypic methods. A single pure colony was selected and sub-cultured to obtain a pure strain.

We used 16s rDNA gene sequencing analysis to identify the genotypic characterization of isolates. Bacterial DNA was extracted from bacterial fluid amplification of culture by using a DP336 kit (Tiangen Biotech Co., Ltd. Beijing, China). PCR was carried out in total volumes of 50 μL containing 25 μL of I-5™ 2×High-Fidelity Master Mix (Tsingke Biological Technology Co., Ltd., Beijing, China), 1 μL of both forward and reverse primers, 1 μL of DNA samples, and 22 μL of sterile distilled water. Amplification of the 16S rDNA was performed using universal primers (27F: 5′-GAG AGT TTG ATC CTG GCT CAG-3′; 1492R: 5′-TAC GGC TAC CTT GTT ACG AC-3′). PCR was performed under the following conditions: the initial denaturation at 95 °C for 3 min, then 39 cycles at 98 °C for 10 s, 55 °C for 15 s, 72 °C for 20 s, and a final extension at 72 °C for 5 min. PCR products (20 μL) were electrophoresed on 1% agarose gel and visualized under a Gel Imager. The PCR product bands were collected from the gel, and purified using a DNA gel recovery kit (Tsingke Biological Technology Co., Ltd., Beijing, China). The sequencing task was accomplished by Tsingke Biological Technology Co., Ltd. The sequences obtained were compared with other selected sequences from the same family strains deposited in the GenBank database by using the Basic Local Alignment Search Tool (BLAST).

### 2.10. Oral Feeding of Cultured Lactobacillus rhamnosus

*Lactobacillusrhamnosus* (*L*. *rhamnosus*) was grown in MRS broth (performed in a 37 °C shaker at 200 r/min for 24 h), and the cultures were harvested and washed with sterile PBS. Subsequently, the concentration of cultures was adjusted to 10^9^ cfu/mL with PBS containing 25% glycerol and stored at −80 °C. Prior to oral feeding, strains were thawed and resuspended in potable water to a concentration of 1 × 10^8^ cfu/mL. Mice were randomly divided into three groups, including the AOM/DSS + *Lac* + BBR group (gavaged with *L*. *rhamnosus* and BBR), the AOM/DSS + BBR group (gavaged with BBR), and the AOM/DSS group (gavaged with potable water). BBR (15 mg/kg/day) and *L*. *rhamnosus* (1 × 10^8^ cfu/mL, 5 days a time) was administrated by the intragastric route for 1 month prior to model induction in the experiment group.

### 2.11. Statistical Analysis

All data are presented as mean ± standard error of the mean (SEM) collected from at least three independent experiments. Differences between two groups were analysed by an unpaired Student’s *t*-test using GraphPad Prism 9.0. One-way analysis of variance (ANOVA) was used for comparisons of more than two groups. Significance was recognised at the value of *p* < 0.05.

## 3. Results

### 3.1. Berberine Precludes Body Weight Loss and Decreases Disease Activity Index (DAI) Score in Dextran Sulfate Sodium Salt (DSS)-Induced Colitis Mice

**Figure 2:**
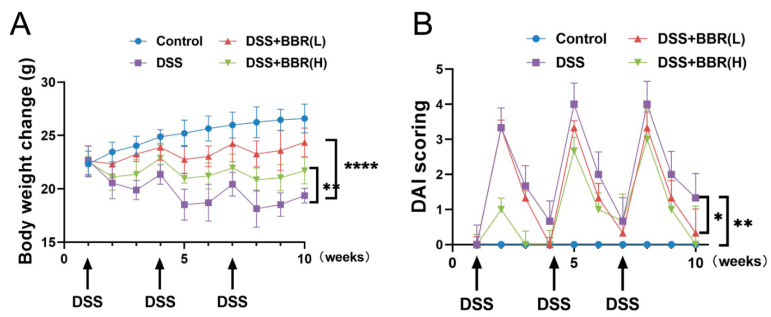
Effects of berberine (BBR) on clinical signs in dextran sulfate sodium salt (DSS)-induced colitis mice. Body weight (**A**) and disease activity index score (**B**) were evaluated weekly at the same time throughout the experimental period (*n* = 10). Data are presented as mean ± SEM of the indicated number of independent experiments. “*” *p* < 0.05, “**” *p* < 0.01, “****” *p* < 0.0001 vs. model group (DSS group).

In order to investigate the effect of BBR on DSS-induced colitis mice, the body weights of mice were examined in each group. The data indicated that a dramatic decrease was observed in body weight after treatment with DSS, and BBR pre-administration reduced body weight losses, especially in the DSS + BBR(H) group (Figure 2A). Furthermore, the mice exhibited a significant increase in disease activity index (DAI) score after DSS treatment, and DAI scores were significantly decreased in the DSS + BBR(L) group and the DSS + BBR(H) group (Figure 2B). Therefore, pre-administration of BBR can prevent weight loss and ameliorate clinical symptoms in DSS-induced colitis mice.

### 3.2. Berberine Inhibits the Expressions of Inflammatory Mediators and Ameliorates Colon Lesions in DSS-Induced Colitis Mice

**Figure 3:**
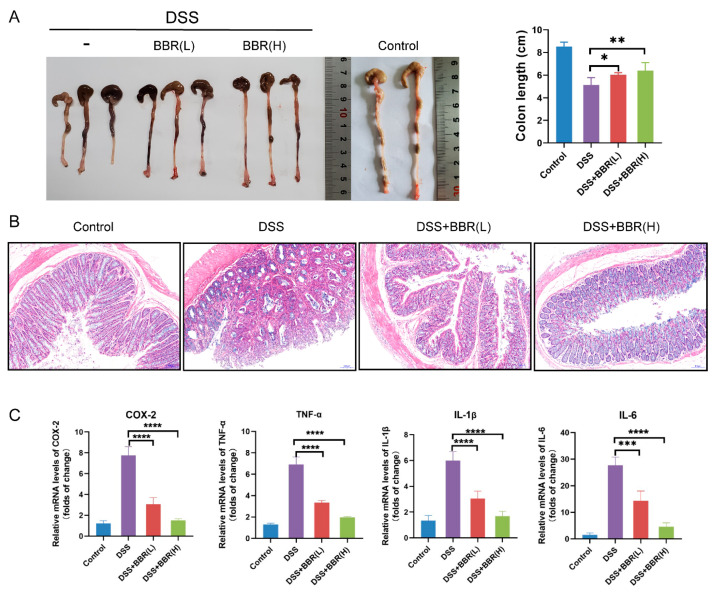
The effect of pre-administration of BBR on DSS-induced colon inflammation. (**A**) Representative photograph and quantitative result showing the length of colon in each group (*n* = 6–10). (**B**) Histopathological changes were examined by hematoxylin-eosin (H&E) staining (*n* = 6–10); scale bars = 50 μm. (**C**) The transcriptional levels of cyclooxygenase (COX-2), tumor necrosis factor (TNF-α), interleukin (IL-1β), and IL-6 in colon tissues detected by quantitative real-time polymerase chain reaction (RT-qPCR). β-actin was used as internal control to normalize target genes transcription, and relative mRNA expression was calculated by the 2^−ΔΔCt^ method (*n* = 5). Data are presented as mean ± SEM of the indicated number of independent experiments. “*” *p* < 0.05, “**” *p* < 0.01, “***” *p* < 0.001, “****” *p* < 0.0001 vs. model group (DSS group).

To investigate the effects of BBR on DSS-induced colon injury, the colons of mice were examined for length measurement. The results indicated that BBR attenuated DSS-induced colon length shortening (Figure 3A). Histological characteristic of colon tissues were analysed after staining with H&E. As shown from the evidence (Figure 3B), a normal intestinal mucosa structure was observed in the control group. In contrast, damaged crypts, irregular distribution of the glands and increased inflammatory cell infiltration were noticed in the DSS stimulation group. Notably, the BBR pre-administration groups showed an obvious improvement in the histological structure of the intestinal mucosa (Figure 3B). Additionally, we further analysed the transcriptional levels of pro-inflammatory cytokines in colon tissues, including COX-2, TNF-α, IL-β, and IL-6. RT-qPCR results showed that the mRNA expression of pro-inflammatory cytokines was significantly increased in the DSS-induced group compared with the model group, but pre-administration with BBR substantially diminished the expression of inflammatory mediators (Figure 3C).

### 3.3. Berberine Inhibits the Development of Azoxymethane (AOM)/DSS-Induced Precancerous Lesions and Improves Intestinal Barrier Function

**Figure 4:**
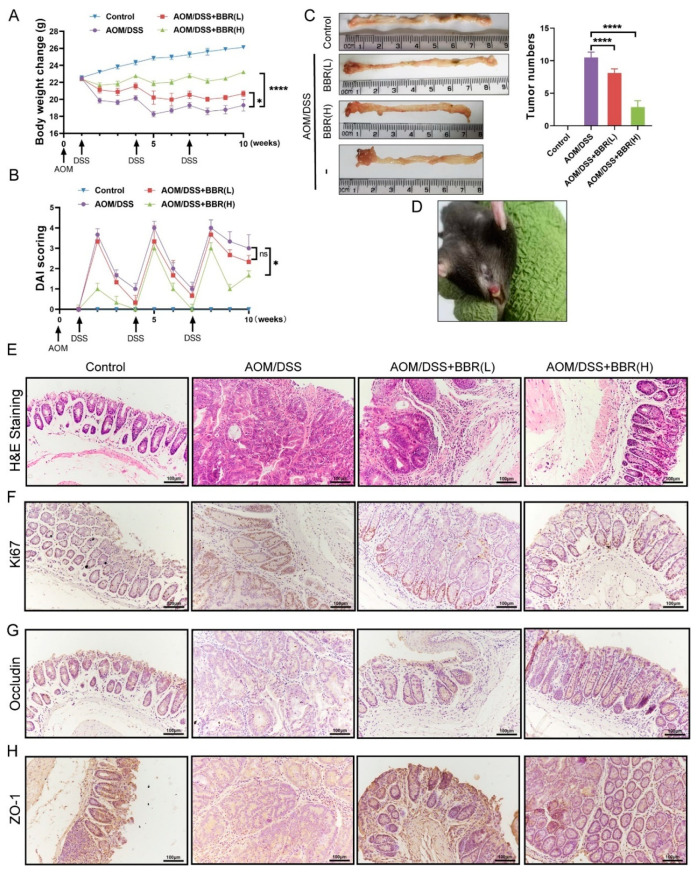
Effects of BBR on azoxymethane (AOM)/DSS-induced precancerous lesions. Body weight (**A**) and disease activity index (DAI) score (**B**) were appraised weekly in each group (*n* = 8). (**C**) Representative gross images of colon tissues and the number of tumors (*n* = 8). (**D**) Mucosal prolapse during defecation in the AOM/DSS-induced group mice. (**E**) Histopathological changes were examined by H&E staining (*n* = 8); scale bars = 100 μm. (**F**) Immunohistochemical staining for Ki67. The expression of ZO-1 (**G**) and Occludin (**H**) in colon tissues were tested by immunohistochemistry; scale bars = 100 μm. Data are presented as mean ± SEM of the indicated number of independent experiments. “*” *p* < 0.05, “****” *p* < 0.0001 vs. model group (AOM/DSS group).

To further identify the effectiveness of BBR on colon cancer, we utilized a mouse model of AOM/DSS-induced colitis-associated carcinogenesis, and extended the period of administration of BBR (30 days). During the experimental period, body weights and DAI scores were appraised weekly in each group. The results showed that body weight losses and DAI scores were significantly increased, and even observed mucosal prolapse during defecation in the AOM/DSS-induced group mice. However, pre-administration of BBR diminished body weight losses and DAI scores, especially in the AOM/DSS + BBR(H) group (Figure 4A–C). In addition, the number of tumors in colon tissues was examined to determine the effects of BBR on tumor formation after AOM/DSS induction. Representative gross images of colon tissues from each group were presented in Figure 4C, and the number of tumors was obviously reduced in the BBR pretreatment group compared with the model group (Figure 4E).

Histopathology analysis revealed that AOM/DSS-induced mice exhibited precancerous lesions, characterized by crypt destruction, inflammatory cell infiltration, and tumor formation, while BBR pre-administration can ameliorate the lesions of the colon (Figure 4F). As a cell-proliferation marker, Ki67 expresses abundantly in malignant tumor tissue. Therefore, the expression of Ki67 in colons were analysed by immunohistochemistry, and the results showed that the expression of Ki67 in the colon was higher in AOM/DSS-induced mice than normal mice. Notably, a decrease in the expression of Ki67 was observed in the BBR pre-administration group (Figure 4F). Additionally, immunohistochemistry staining for occludin and ZO-1 exhibited enhanced immunoreactivity in the BBR-treated group compared with the AOM/DSS-induced group (Figure 4G).

### 3.4. Berberine Exhibits Anti-Cancer Activity via the JNK/STAT3 and β-Catenin Pathways in AOM/DSS-Induced Colitis-Associated Carcinogenesis Mice

**Figure 5:**
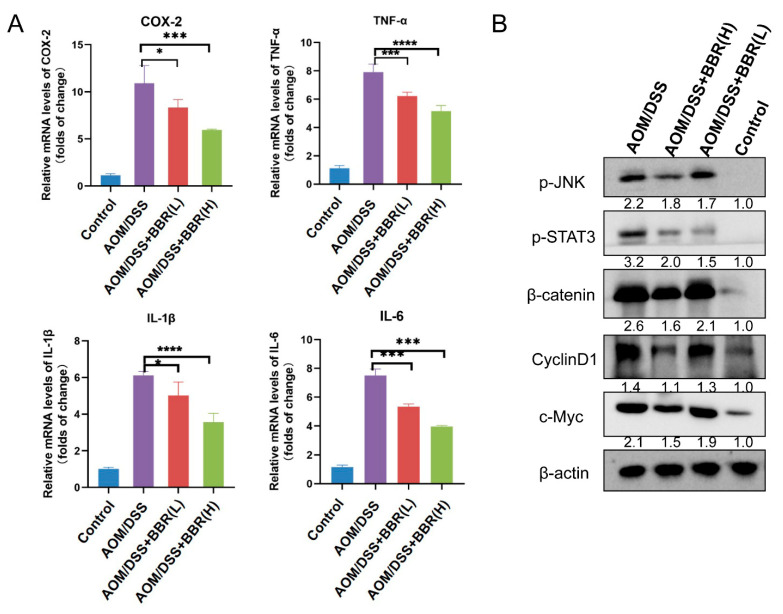
Detection of inflammatory factors in colon tumor tissue. (**A**) The expression levels of inflammatory factors COX-2, TNF-α, IL-1β and IL-6 in colon tissues detected by RT-qPCR. β-actin was used as internal control to normalize target gene transcription, and relative mRNA expression was calculated by the 2^−ΔΔCt^ method (*n* = 5). (**B**) The protein expression of p-JNK, p-STAT3, β-catenin, CylinD1 and c-Myc were examined by Western blot analysis. β-actin was used as the loading control. Relative expression of proteins was quantified using ImageJ and expressed as a ratio. Data are presented as mean ± SEM of the indicated number of independent experiments. “*” *p* < 0.05, “***” *p* <0.001, “****” *p* < 0.0001 vs. model group (AOM/DSS group).

Chronic inflammation plays pivotal role in the development and progression of colon cancer. The transcriptional levels of pro-inflammatory cytokines were detected by RT-qPCR. The results showed that increased expression of IL-6, TNF-α, IL-1β, and COX-2 was found in the AOM/DSS-induced group, but they were dramatically diminished after BBR pre-administration (Figure 5A). To identify the anti-inflammatory mechanism of BBR, the translational levels of critical pathway proteins were examined by Western blot analysis. As shown in Figure 5B, the protein expression of p-JNK and p-STAT3 was markedly increased in the AOM/DSS-induced group compared with the control group, while their expression levels were restrained by pre-administration of BBR. In addition, we observed that pre-administration of BBR reduced β-catenin expression compared with the AOM/DSS-induced group, which synchronously caused a decrease in the expression of c-Myc and cyclinD1 (Figure 5B).

### 3.5. Berberine Modulates AOM/DSS-Induced Gut Microbiota Dysbiosis

**Figure 6:**
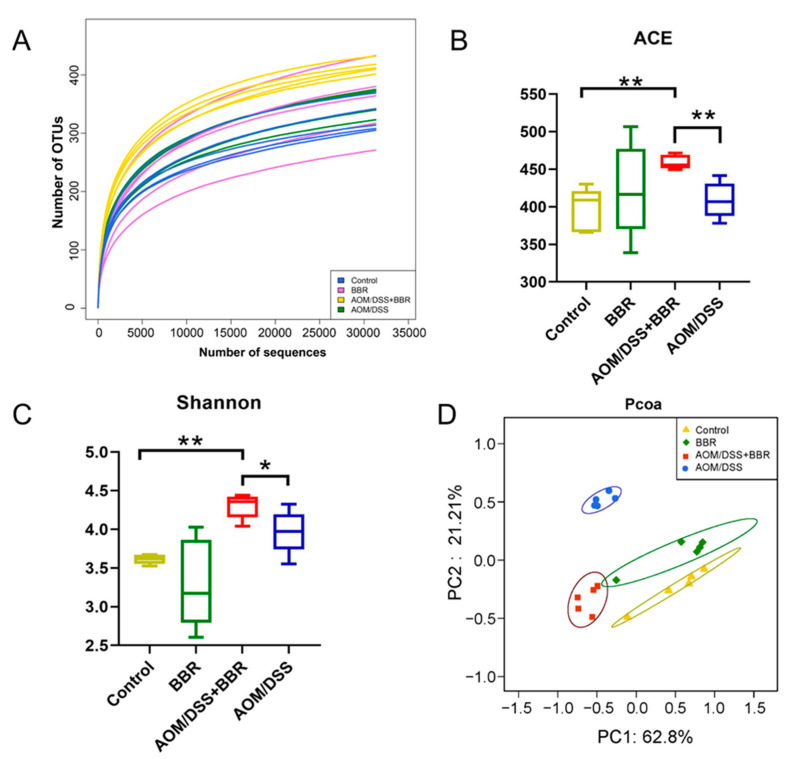
Analysis of gut microbiota richness and diversity in the Control, AOM/DSS, BBR, and AOM/DSS + BBR groups. (**A**) The rarefaction curves show the numbers of unique operating taxonomic units (OTUs) in each group (*n* = 5). The ACE index (**B**) and Shannon (**C**) index of fecal samples in each group (*n* = 5). (**D**) Weighted UniFrac principal coordinate analysis (PCoA) based on OTU abundance (*n* = 5). Data are presented as mean ± SEM of the indicated number of independent experiments. * indicates *p* < 0.05, ** indicates *p* < 0.01.

Dysbiosis of gut microbiota is a pivotal characteristic of CRC. In an attempt to observe the effects of BBR on the gut microbiota, the gut microbiota composition was revealed by 16S rRNA gene sequencing in fecal samples. As shown in Figure 6A, the curve tend to be smooth, which indicates that the sequencing depth is adequate. The ACE index and Shannon index have been used to reflect the species richness and diversity of the community, respectively. Our results showed that both ACE richness index and Shannon diversity index were significantly higher in the AOM/DSS + BBR group than that in the AOM/DSS-induced group (Figure 6B,C). The weighted UniFrac principal component analysis (PCoA) was employed to estimate the phylogenetic similarity and difference of gut microbiota composition in each group. In the coordinate graph, the closer the distance between the two samples the more similar the species composition and structure of the two samples. The PCoA result revealed that the composition and structure of gut microbiota from the AOM/DSS-induced group was obviously different from the other groups, suggesting the homeostasis of gut microbiota was dramatically disrupted by the AOM/DSS stimulus (Figure 6D). Notably, a closer distance between the AOM/DSS + BBR group and the control group was observed, suggesting that pre-administration of BBR can partially alleviate the gut microbiota dysbiosis induced by the AOM/DSS stimulus.

**Figure 7:**
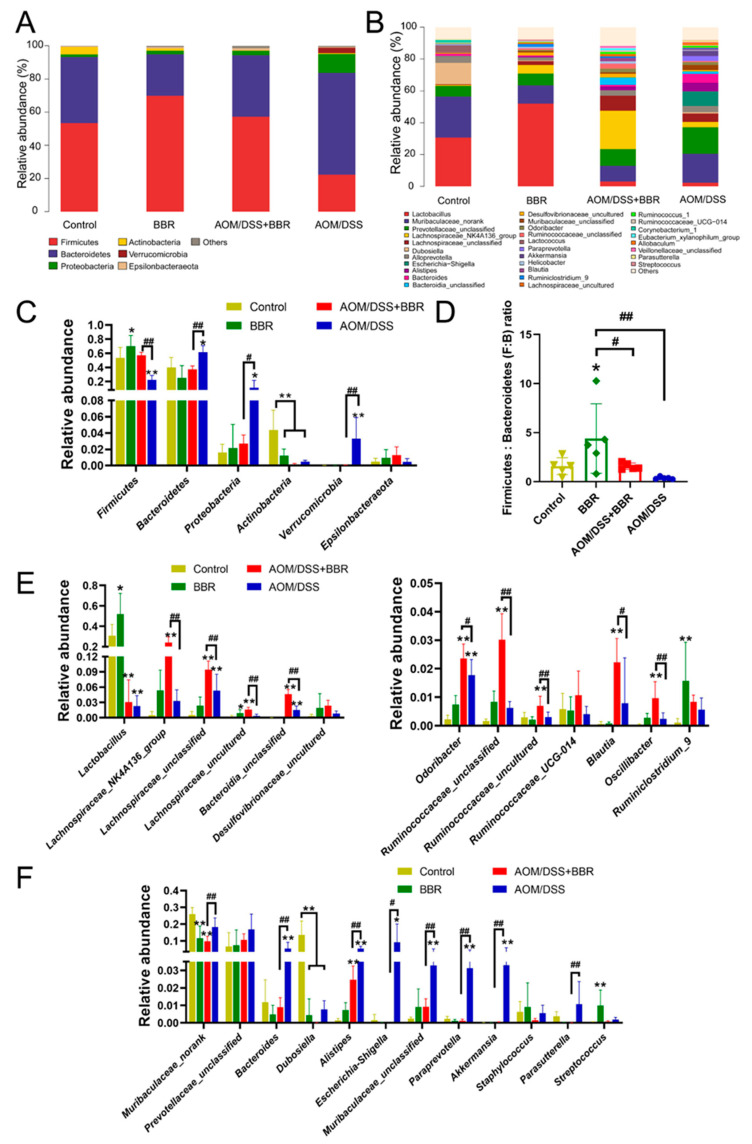
The effect of BBR on gut microbiota composition. The gut microbiota composition profiles at the phylum (**A**) and genus (**B**) level. Less abundant bacteria are grouped under the category “Other”, (*n* = 5). (**C**) Relative abundance of gut microbiota at the phylum level (*n* = 5). (**D**) The phylum Firmicutes: Bacteroidetes (F:B) ratio in each group. (**E**) Up-regulated gut microbiota in the AOM/DSS + BBR group but low in the AOM/DSS group (*n* = 5). (**F**) Down-regulated gut microbiota in the AOM/DSS + BBR group but high in the AOM/DSS group (*n* = 5). Data are presented as mean ± SEM of the indicated number of independent experiments. * *p* < 0.05, ** *p* < 0.01 vs. Control group, ^#^ *p* < 0.05, ^##^ *p* < 0.01 vs. AOM/DSS group.

The gut microbiota taxa and their relative abundance were analysed to further identify the species that caused significant inter-group differences. Hence, we investigated the taxonomic composition at the phylum and genus levels. As shown in Figure 7A, the taxonomic composition at the phylum level mainly contained Firmicutes, Bacteroidetes, Proteobacteria, Actinobacteria, Verrucomicrobia, and Epsilonbacteraeota. Among them, Firmicutes, Bacteroidetes, Proteobacteria, and Actinobacteria were the most abundant phyla. The relative abundance of Firmicutes and Actinobacteria was diminished in the AOM/DSS-induced group in comparison to the control group, while AOM/DSS stimulus increased the abundance of Bacteroidetes, Proteobacteria, and Verrucomicrobia. Notably, pre-administration of BBR can mitigate the variation of gut microbiota caused by AOM/DSS stimulation (Figure 7A,C). Additionally, The Firmicutes/Bacteroidetes (F:M) ratio in the BBR group was significantly lower than that in the control group, and pre-administration of BBR increased the F:M ratio compared with the AOM/DSS-induced group (Figure 7D). At the genus level, an increased abundance of *Lactobacillus*, *Lachnospiraceae_NK4A136_group*, *Odoribacter*, *Ruminococcaceae_UCG-014*, *Blautia*, *Oscillibacter*, *Ruminiclostridium_9*, and some uncultured or unclassified genera belonging to Lachnospiraceae, Bacteroidia, Desulfovibrionaceae, and Ruminococcaceae in the AOM/DSS + BBR group compared with the AOM/DSS group (Figure 7E). Also noteworthy is an obvious reduction in the abundance of *Muribaculaceae_norank*, *Bacteroides*, *Dubosiella*, *Alistipes*, *Escherichia-Shigella*, *Parasutterella*, *Akkermansia*, *Paraprevotella*, *Staphylococcus*, and unclassified genera from Prevotellaceae and Muribaculaceae (Figure 7F).

### 3.6. Lactobacillus rhamnosus (L. rhamnosus) Treatment Improves the Anti-Cancer Effect of BBR

**Figure 8:**
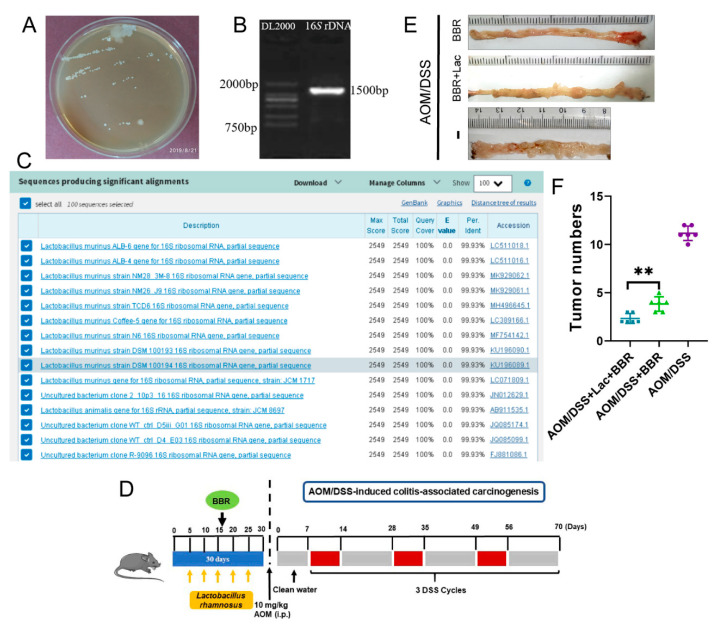
*L. rhamnosus* can promote the preventive effect of BBR on inflammatory colorectal cancer. (**A**) Screening of *Lactobacillus* by MRS bacterial medium. (**B**) PCR amplification and sequencing of bacterial 16S rDNA region. (**C**) Results of sequence homologous alignment in NCBI database. (**D**) Schematic diagram of bacterial solution, BBR intragastric administration and AOM/DSS treatment. (**E**) Representative gross images of colon tissues in each group (*n* = 6). (**F**) Quantitative result showing the number of tumors in each group (*n* = 6). Data are presented as mean ± SEM of the indicated number of independent experiments. ** indicates *p* < 0.01.

According to the results from 16S rRNA gene sequencing, *Lactobacillus* was a dominant genus accounting for more than 30% of total gut bacteria, and BBR pre-administration dramatically increased the abundance of *Lactobacillus* (Figure 7B,C). Consequently, we hypothesized whether the presence of *Lactobacillus* is essential for improving the anti-cancer activity of BBR. In order to further identify a specific bacterium that exerted beneficial effects on AOM/BSS-induced colitis-associated carcinogenesis mice, *Lactobacillus* were preliminarily isolated and screened using MRS medium (Figure 8A). Finally, the isolate was identified as *L. rhamnosus* by 16S rDNA sequencing analysis (Figure 8B,C).

In order to further investigate whether *L. rhamnosus* plays an instrumental role in the therapeutic action exerted by BBR, BBR or/and *L. rhamnosus* were administrated by the intragastric route (Figure 8D). The results indicated that oral administration of *L. rhamnosus* and BBR dramatically diminished the number of tumors in comparison to the AOM/DSS + BBR group (Figure 8E,F). Therefore, *L. rhamnosus* is an important probiotic for enhancing the beneficial effects of BBR in the prevention of AOM/BSS-induced colitis-associated carcinogenesis.

## 4. Discussion

As a double-edged sword, inflammation mediates a protective response against pathogen infection and tissue damage, while chronic inflammatory conditions also trigger carcinogenic events, especially those involved in the tumorigenesis of gastrointestinal organs [3,4]. The increasing disease burden caused by CRC has become one of the major public health problems all over the globe [38,39,40]. Tumorigenesis has been considered to be a sophisticated biological process, and chronic inflammatory conditions are indispensable in the initiation of CRC. In recent years, immune modulators and nonsteroidal anti-inflammatory drugs (NSAIDs) have been broadly used for CRC therapy, whereas several side effects have been found during the therapeutic process [41,42]. Therefore, many efforts have been devoted to excavate novel preventive therapeutics, and chemopreventive agents with anti-inflammatory and anti-tumourigenic benefits have attracted great attention, especially as safe and inexpensive compounds derived from foods and herbs [18,19,20,21,43].

BBR is an active ingredient extracted from plants, which has aroused considerable attention due to its multiple biological activities [23,24,25]. Available evidence suggests that BBR exhibits enormous therapeutic potential in various diseases, and the underlying mechanism is involved in the modulation of inflammation, oxidation, autophagy, and intestinal microbiota [25,31,44]. Importantly, the anti-cancer activity of BBR has been extensively investigated in both preclinical models and clinical trials, particularly in gastrointestinal cancers [30,31,32,33,34]. These previous studies strongly suggest that BBR could be used as a promising anti-cancer agent for cancer treatment and prevention. However, whether prophylactic BBR administration also exerts beneficial effects in the development of cancers is still poorly understood. Here, we analysed the effects of pre-administration of BBR in DSS-induced colitis and AOM/DSS-induced colitis-associated carcinogenesis mice based on body weight, disease activity index (DAI) score, and colon histology. We found that pre-administration of BBR can preclude body weight losses, palliate clinical signs, and ameliorate colon lesions.

Previous studies have reported that soluble mediators play a crucial role in perpetuating a chronic inflammatory microenvironment, including IL-6, IL-1β, TNF-a, COX-2 and PGE_2_, which are propitious to promoting CRC cell angiogenesis, growth, and migration/invasion [32,45]. Therefore, the blockade of these cytokines contributes to prevent CRC progression. Wang and his colleagues found that depletion of neutrophil or blockade of IL-1β activity significantly reduced mucosal damage and tumor formation in a colitis-associated cancer (CAC) mice model [46]. Additionally, inhibition of IL-6 and anti-TNF-α therapy also presented therapeutic benefits in CRC clinical trials [47,48]. The data from our study indicated that the mRNA levels of IL-6, IL-1β, TNF-a, and COX-2 in colon increased significantly after DSS or AOM/DSS treatment, whereas pre-administration of BBR decreased the transcriptional levels of inflammatory cytokines, suggesting BBR could inhibit inflammatory responses. These findings are consistent with previous studies, in which the administration of BBR suppressed the secretion of IL-1, IL-1β, IL-6, IL-12, TNF-α, TGF-β and IFN-γ in a DSS-induced ulcerative colitis rat model [26]. In this research, BBR was administrated regularly by the oral route prior to the establishment of the disease model, suggesting that BBR provided chemonpreventive effects against inflammation and tumorigenesis. Taken together, inflammatory cytokines are closely associated with tumor development and progression, and BBR is able to target inflammation for the prevention and treatment of CRC and other inflammation-associated cancers.

Transcription factors are indispensable for signaling by inflammatory mediators during intestinal inflammation, including NF-κB and STATs, which ultimately contribute to the development and progression of CRC. The NF-κB signaling is activated by various inflammatory molecules IL-1β and TNF-α; simultaneously, the activation of NF-κB can enhance the secretion of pro-inflammatory cytokines themselves, which is responsible for the presence of chronic inflammatory conditions [49]. Moreover, signal transducer and activator of transcription 3 (STAT3) also plays a crucial role in inflammation-associated tumorigenesis because of its pro-inflammatory and oncogenic properties. According to the data gathered from several studies, the JNK/STAT3 signaling pathway is activated in the progression of various tumors, such as lung cancer, breast cancer, and CRC [32,50,51]. Similarly, the activation of JNK/STAT3 signaling was observed in the colon tissues of the AOM/DSS-induced group, and pre-administration of BBR suppressed the expression of p-JNK and p-STAT3, suggesting BBR may be involved in blockading JNK/STAT3 signaling. Additionally, Wnt/β-catenin signaling has also been demonstrated to be a key regulator of inflammatory signaling, which is conducive for tumorigenesis. The accumulation of β-catenin protein is a common feature of several cancers, such as breast cancer, gastrointestinal cancers, hepatocellular carcinoma, endometrial cancer, and ovarian cancer [10]. In the absence of Wnt ligands, β-catenin is phosphorylated and targeted for degradation, while Wnts bind to their receptors leading to its accumulation and translocation into the nucleus, where it combines with Tcf/Lef to stimulate the transcription of genes involved in the development and progression of cancer, such as COX-2, VEGF, survivin, c-Myc and cyclinD1 [52,53]. Likewise, our results showed that increased expression of β-catenin was noticed in the colons of model mice, while the inhibitory effect of BBR on β-catenin and the expression of the Wnt target genes c-Myc and cyclinD1. Taken together, this evidence indicated that BBR exerts anti-inflammatory and cancer preventive activity possibly through the modulation of JNK/STAT3 and β-catenin signaling.

As a pivotal constituent of the intestinal tract, the gut microecosystem is essential for modulating intestinal homeostasis and host health due to its protective capability for mucosal barrier, metabolism of nutrition, and immunity [5,54]. Accumulating evidence based on metagenomics and experimental models has verified the potential influences of gut microbiota in modulating CRC development [11,55]. Recently, growing evidence demonstrated that BBR may contribute to maintaining the gut homeostasis by modulating gut microbiota and thereby exhibits health-related benefits in various diseases, including obesity, atherosclerosis, diabetes, inflammatory disease, and cancer [14,35,56,57]. Hence, we investigated the effects of BBR on gut microbiota via microbial sequence analyses. Our results indicated that compared with the AOM/DSS-induced group, a significant decrease was noticed in the abundances of the phyla Bacteroidetes, Proteobacteria, and Verrucomicrobia in the AOM/DSS + BBR group; meanwhile, the abundance of the *Firmicutes* phylum was increased in the AOM/DSS + BBR group. Indeed, Bacteroidetes, Proteobacteria, and Verrucomicrobia have been demonstrated to play a pivotal role in the activation of inflammation, whereas Firmicutes can produce butyric acid, which is instrumental in protecting the intestinal wall and suppressing intestinal inflammation and CRC incidence [11,58,59]. Additionally, the ratio of Firmicutes to Bacteroidetes is regarded as a relevant biomarker of gut dysbiosis, and it is low in IBD patients but high in obese patients [60,61,62]. Similarly, our results showed that the ratio was decreased in the AOM/DSS-induced group and pre-administration of BBR can restore the ratio of Firmicutes to Bacteroidetes.

Dysbiosis of the gut microbiome disrupts intestinal homeostasis due to the diversity of gut microbiota and its complex interaction with the intestine. Importantly, increasing evidence suggests that the gut microbiota and its metabolites are involved in the development and progression of several types of cancer by influencing inflammation, DNA damage, and apoptosis [63]. Several studies have identified that the decrease of several potentially beneficial bacterial species (including *Lactobacillus*, *Bifidobacterium*, *Oscillibacter*, *Ruminiclostridium 9*, and *Dubosiella*), and the increase of some adverse bacterial species (including *Enterococcus*, Enterotoxigenic *bacteroides fragilis*, *Streptococcus*, *Helicobacter*, *Fusobacterium nucleatum*, *Escherichia-Shigella*, *Klebsiella* and *Akkermansia*), which are closely correlated with an increased risk of CRC [11,13,17,64,65,66]. The data from our study showed that AOM/DSS stimulus inevitably disturbed the balance of intestinal microbiota, as shown by a dramatic decrease in the abundance of dominant microbiota *Lactobacillus* and *Dubosiella*, and an increase in the abundance of harmful bacteria *Bacteroides*, *Escherichia-Shigella*, and *Akkermansia*. Conversely, pre-administration of BBR normalized these bacteria to relatively normal levels, suggesting BBR plays an important role in modulating gut microbiota homeostasis. Additionally, some of the butyrate-producing bacteria, belonging to the Lachnospiraceae and Ruminococcaceae families, which have increased dramatically after BBR prointervention. Available evidence has demonstrated that butyrate can enhance intestinal epithelial barrier function and modulate the intestinal immune response [63,67]. Strikingly, a dramatic increase in the abundance of *Lactobacillus* was observed after pre-administration of BBR, but there was no significant difference between the AOM/DSS and AOM/DSS + BBR group. In this scenario, BBR-induced increase in *Lactobacillus* could be harnessed to improve the harsh pathological environments. Cumulative research has indicated that *Lactobacillus,* as a recognized probiotic, can rehabilitate intestinal homeostasis in gastrointestinal inflammatory diseases [65,68]. Moreover, *L. rhamnosus* treatment can reduce the expression of β-catenin and the inflammatory proteins NFκB-p65, COX-2 and TNF-α in dimethyl hydrazine (DMH)-induced colon carcinogenesis [69]. Similarly, our results imply that *L. rhamnosus* pretreatment increased the protective effects of BBR in AOM/DSS-induced colitis-associated carcinogenesis, suggesting that *L. rhamnosus* plays an instrumental role in preventing colon carcinogenesis. Altogether, the anti-cancer activity of BBR is in part explained by its role in the modulation of the gut microbiota.

As described in previous studies, BBR has been validated as a multifunctional natural product with diverse therapeutic applications. Similarly, our results indicate that pre-administration of BBR exerts cancer-preventive effects by modulating inflammation and gut microbiota composition. However, BBR-mediated inhibition of the inflammatory response whether depends on the complex interactions between the microbiota and the host is unclear. Recent findings suggested that BBR might modify bacterial metabolites and increase the levels of butyrate and glutamine to reduce inflammation in the intestine [14]. Additionally, dysbiosis induces secondary bile acid deficiency in inflammatory-prone UC patients, which aggravates the pro-inflammatory state [70]. Therefore, a better fundamental understanding the roles of microbiota in colorectal carcinogenesis based on ecology and physiology is required, which would highlight the potential contribution of gut microbiota to the multifunctional bioactivity of BBR.

## 5. Conclusions

**Figure 9:**
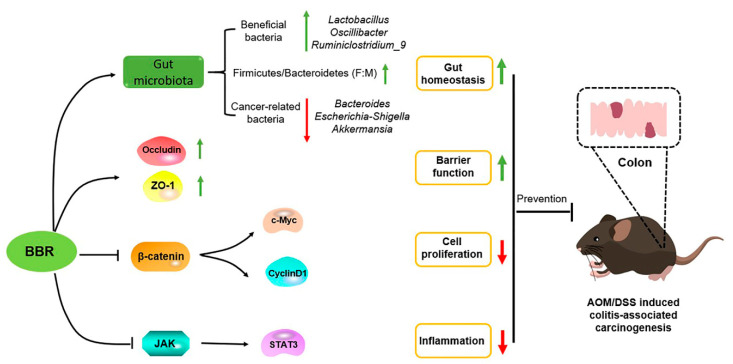
The mechanism underlying of BBR in prevention of AOM/DSS-induced colitis-associated carcinogenesis. BBR exerts anti-cancer activity via inhibiting inflammation and tumor cell proliferation, and enhancing intestinal barrier function, as well as maintaining gut homeostasis.

The treatment of cancers is a major medical challenge facing humanity due to their complex pathological mechanisms. Therefore, chemoprevention with multifunctional natural products is the most practical strategy to preclude carcinogenic progression, and thus contribute to reduce the morbidity and mortality of cancers. Pre-administration of BBR exhibits cancer-preventive effects in AOM/DSS-induced colitis-associated carcinogenesis model mice. The mechanism underlying such an effect was involved in inhibiting inflammation and tumor cell proliferation, and enhancing intestinal barrier function, as well as maintaining gut homeostasis (Figure 9). As a consequence, BBR might be a promising chemopreventive agent for the prevention of CRC, and a supplement of BBR in a normal diet could confer upon the organism health-related benefits.

## Data Availability

The datasets generated during and/or analysed during the current study are available from the corresponding author on reasonable request.

## Abbreviations

AOM	Azoxymethane
BBR	Berbrine
CD	Crohn’s disease
COX-2	cyclooxygenase-2
CRC	colorectal cancer
DAI	disease activity index
DSS	dextran sodium sulfate
IBD	inflammatory bowel disease
IL	interleukin
MRS	de Man Rogosa Sharpe
NO	nitric oxide
PG	prostaglandin
TNF-α	tumor necrosis factor-alpha
UC	ulcerative colitis
